# Editorial: Brain-Metabolic Crossroads in Severe Mental Disorders—Focus on Metabolic Syndrome

**DOI:** 10.3389/fpsyt.2019.00492

**Published:** 2019-07-12

**Authors:** Virginio Salvi, Tomas Hajek

**Affiliations:** ^1^Department of Neuroscience, ASST Fatebenefratelli Sacco, Milan, Italy; ^2^Department of Psychiatry, Dalhousie University, Halifax, NS, Canada

**Keywords:** insulin, metabolic syndrome (MetS), cognition, metabolism, brain

A large body of evidence shows that patients with severe mental illnesses are more frequently affected by metabolic disorders such as diabetes and the metabolic syndrome, which contribute to cardiovascular morbidity and reduced life expectancy (1). In addition, we are learning more about the effects of metabolic pathways and key metabolic hormones on the functioning of the brain. For example, even though insulin is not necessary for the neuronal uptake and utilization of glucose in the brain, it has multiple roles in central nervous system, from acting as a growth factor to directly influencing cognitive functions such as memory formation (2). It is thus not surprising that patients with type 2 diabetes display cognitive impairments, even in the absence of macrovascular complications. They are at an increased risk for accelerated brain aging and dementia. Furthermore, diabetes in major psychiatric disorders is not only associated with worse somatic health, but also with poor psychiatric outcomes, poor response to psychiatric medications, and with greater extent of neurostructural and neurochemical brain alterations (3, 4).

Besides insulin, other mediators such as total and HDL cholesterol levels play key roles in the brain and were found to have pathoplastic effects on psychiatric disorders: the former has been repeatedly associated with suicide risk (5), the latter with higher prevalence of negative symptoms in schizophrenia (6). Considering the role of these metabolic pathways on the functioning of the brain, it is not surprising that metabolic syndrome, which is highly prevalent in patients with bipolar disorders and schizophrenia (7) and tends to increase over time (8), contributes to cognitive decline (9) and brain ageing (10) in schizophrenia and even interferes with the efficacy of cognitive remediation therapy (11).

The scope of the present Research Topic, then, was to collect papers investigating the complex interplay between brain/cognitive/psychopathological and metabolic disturbances in severe mental disorders. Several eminent researchers contributed to this collection, which includes seven original research articles and three literature reviews. These studies contribute to our knowledge about the interrelationship between cognitive and metabolic disturbances in severe mental illnesses. They highlight the need to rethink psychiatry as a branch of medicine deeply intertwined with physical health.

The observation that severe mental illnesses are associated with adverse metabolic profiles draws back to the pre-pharmacological era. As far back as in late 19^th^ century, the British psychiatrist Henry Maudsley noted that “diabetes is a disease that often shows itself in families where insanity prevails” (12). More than one century later the association is further confirmed, among others, by the work of Toma et al., who reported a more frequent family history of cardiovascular disease in families of subjects with bipolar disorder than in families of healthy controls. Moreover, the authors reported for the first time that the risk of cardiovascular disease increased in families with a family history for bipolar disorder, suggesting common pathogenetic pathways to be investigated in genetic studies.

Besides the common genetic factors, the comorbidity between mental and metabolic disorders may also be explained by common environmental factors. Stress can both influence the expression and severity of mental disorders and contribute to the development of insulin resistance and metabolic syndrome. To address this, Sun et al. conducted a cutting-edge study correlating levels of childhood abuse with glucose metabolism and resting-state functional connectivity in a sample of overweight adolescents with depression. The authors found that high level of abuse was associated with dysfunctional connectivity between amygdala, precuneus, and nucleus accumbens. This connectivity pattern was, in turn, associated with decreased levels of glycemia and insulinemia, possibly reflecting a compensatory response in early age. Interestingly, other studies found association between insulin resistance and early life trauma in depressed individuals (13), so we need more research to clarify the existence and directions of these associations and the factors that may mediate/moderate them.

In another article by Knytl et al., cortisol and other neurosteroids did not qualify as endophenotypes in schizophrenia: although cognitive functions were impaired in patients versus siblings and controls, stress hormone cortisol and other steroids were neither different between the groups nor found to mediate the association with cognitive function. It should be noted, however, that the high number of comparisons and the small sample size lowered the statistical power. It would still be interesting to investigate the effects of neurosteroids on cognition in larger samples.

Increased stress hormones, pro-inflammatory mediators, and alterations in insulin signaling, seem nevertheless to convey increased risk for both metabolic and mental disorders, as Lyra e Silva and colleagues comprehensively describe in their review. The authors concluded their work by discussing which medications used to treat metabolic disorders also exert positive effects on cognition or psychopathology. To this end, liraglutide, an agonist at GLP-1 receptor used to control type 2 diabetes, is one of the most promising drugs. Liraglutide improved overall cognitive performances in a group of subjects with mood disorders (14), and several trials testing its effect on Alzheimer’s dementia are ongoing. In this Research Topic, Cuomo et al. evaluated its efficacy in reducing weight in severely obese patients with depressive and bipolar disorders. In their study, liraglutide was effective in 50% of the treated patients, who lost an average of 10 kg during the 6 months of treatment. Since liraglutide is still a very expensive antidiabetic medication, studying whether it also has pro-cognitive or pathoplastic effects might create an even stronger impetus for its use in participants with severe mental disorders.

Aside from these other factors, antipsychotic medications are among the strongest contributors to weight gain and diabetes in subjects with psychiatric illnesses. Some antipsychotics were also thought to contribute to worse cognition in patients with schizophrenia (15), although it is challenging to disentangle the effects of medications from the effects of illness. The review by Mackenzie et al. comprehensively summarizes the interplay between antipsychotic use, metabolic effects, and cognition in schizophrenia. Early in the course of illness, antipsychotics seem to convey benefits to cognition by countering the detrimental effects of mental disorders. However, in the long run, as their effect on metabolism grows, their continuous use may start to negatively affect cognitive functioning.

Attempts to reduce the metabolic impact of antipsychotics are thus warranted. In their retrospective analysis of a cohort of veterans, Chipchura et al. assessed for the first time the impact of time of administration on the metabolic profile of aripiprazole. Interestingly, nighttime administration led to a worse metabolic profile in terms of reduced HDL cholesterol. According to the authors, the effect might be mediated by the blocking of pancreatic D2/D3 receptors, leading to a disinhibition of insulin release during nighttime, eventually reducing lipolysis and altering the lipid profile.

In contrast to antipsychotics, antidepressant medications have usually been regarded as neutral in their effects on metabolism. The review by Gramaglia et al. indeed does not find an association between antidepressants and metabolic syndrome, with the notable exception of anti-histaminergic antidepressants such as the tricyclics or mirtazapine, which had previously been associated with abdominal obesity and reduction in HDL levels (16).

The impact of treatment on metabolic markers has been extensively studied. More recently, researches have started to examine how metabolic disorders can affect the psychopathological or cognitive outcomes in major mental disorders. For instance, insulin resistance is associated with resistance to treatment with lithium salts (17). In keeping with this trend, Soontornniyomkij et al. showed that in schizophrenia, insulin resistance was associated with a preponderance of negative symptoms. In a large sample of suicide attempters, Aguglia et al. added further evidence about the association between low total cholesterol blood levels and suicide attempts. For the first time, they also showed that low total cholesterol may be linked with a higher lethality of the suicide attempt. This may perhaps be related to the reduction of serotonin transporters in subjects with low cholesterol levels, eventually resulting in higher levels of impulsive behaviors.

Taken together, the high-quality contributions gathered in this Research Topic covered several aspects of the reciprocal influences between metabolism and cognition/psychopathology in severe mental illness, and constitute a step ahead in this intriguing and expanding area of research.

## Author Contributions

VS and TH equally contributed to designing and writing the manuscript, and approved it for publication.

## Funding

TH was supported by grants from the Canadian Institutes of Health Research (grant number 142255); the Nova Scotia Health Research Foundation; Brain and Behavioral Research Foundation grant to TH; the Ministry of Health, Czech Republic (grant number 16-32791A).

## Conflict of Interest Statement

The authors declare that the research was conducted in the absence of any commercial or financial relationships that could be construed as a potential conflict of interest.
